# Feeding Delivery of dsHvSnf7 Is a Promising Method for Management of the Pest *Henosepilachna* *vigintioctopunctata* (Coleoptera: Coccinellidae)

**DOI:** 10.3390/insects11010034

**Published:** 2019-12-31

**Authors:** Jing Lü, Zhuoqi Liu, Wei Guo, Mujuan Guo, Shimin Chen, Huali Li, Chunxiao Yang, Youjun Zhang, Huipeng Pan

**Affiliations:** 1 Key Laboratory of Bio-Pesticide Innovation and Application, South China Agricultural University, Guangdong Province, Guangzhou 510642, China; 13556143025@stu.scau.edu.cn (J.L.); liuzhuoqi@stu.scau.edu.cn (Z.L.); gwei8290@stu.scau.edu.cn (W.G.); 13265915482@stu.scau.edu.cn (M.G.); smchen@stu.scau.edu.cn (S.C.); 2 Engineering Research Center of Biocontrol, Ministry of Education and South China Agricultural University, Guangdong Province, Guangzhou 510642, China; 3 State Key Laboratory for Conservation and Utilization of Subtropical Agro-bioresources, South China Agricultural University, Guangzhou 510642, China; hualili@scau.edu.cn (H.L.); yangchunxiao@scau.edu.cn (C.Y.); 4 Department of Plant Protection, Institute of Vegetables and Flowers, Chinese Academy of Agricultural Sciences, Beijing 100081, China

**Keywords:** *Henosepilachna vigintioctopunctata*, *HvSnf7*, feeding RNAi, mortality, ultrastructural change

## Abstract

RNA interference (RNAi) techniques have emerged as powerful tools in the development of novel management strategies for the control of insect pests, such as *Henosepilachna vigintioctopunctata*, which is a major solanaceous pest in Asia. Our results showed that levels of *HvSnf7* expression were greater in larval midguts than in other tissues. Silencing of *HvSnf7* led to greater *H. vigintioctopunctata* mortality rates and appeared to be time- and partially dose-dependent. Bacterially expressed dsHvSnf7 that was applied to detached plant leaves caused 98, 88, and 60% mortality in 1st and 3rd instars, and adults after 10, 12, and 14 d, respectively; when applied to living plants, bacterially expressed dsHvSnf7 led to mortality in 1st and 3rd instars, with no effect on adults. Bacterially expressed dsHvSnf7 led to improved plant protection against *H. vigintioctopunctata*. Ultrastructural changes caused by HvSnf7-RNAi in larval midguts showed extensive loss of cellular contents that indicate loss of membrane integrity. This study indicate that *HvSnf7* potentially can be used as RNAi target gene for controlling of *H. vigintioctopunctata*.

## 1. Introduction

RNA interference (RNAi) is a sequence-specific method for the inhibition of target gene expression that may be used in the exploitation of species-specific and sustainable insect pest control [1,2,3,4]. RNAi-based products are expected to reach the market in the form of transgenic plants and non-transgenic alternatives, such as the spray application of double-strand RNA (dsRNA) to crops to induce silencing of specific genes [5,6]. In the past decade, a number of studies have been published on the application of RNAi for the control of pests from a wide range of insect orders, including the Coleoptera, Diptera, Hemiptera, Lepidoptera, and Orthoptera [7,8,9,10,11,12,13,14]; coleopterans, such as the western corn rootworm (*Diabrotica virgifera virgifera*) [7], the red flour beetle (*Tribolium castaneum*), and Colorado potato beetle (*Leptinotarsa decemlineata*) [15] have been shown to be highly susceptible to dsRNAs.

*Henosepilachna vigintioctopunctata* (Fabricius) (Coleoptera: Coccinellidae) is one of the most economically important insect pests in Asia, causing significant damage to vegetables, particularly those in the Solanaceae [16,17]. Currently, this species is mainly controlled by chemical pesticides [18]; however, against the background of the requirement for negative growth of pesticide use in China, there is an urgent need to develop non-chemical pest-specific alternatives with new modes of action for the control of *H. vigintioctopunctata*.

Endosomal sorting complexes required for transport (ESCRTs), which were first discovered in yeast, are required in multivesicular body (MVB) biogenesis [19], and as such, the ESCRT pathway is a key regulator of biological processes important for eukaryotic cell growth and survival. In yeast, humans, and insects, the ESCRT pathway is composed of five distinct, highly conserved sub-complexes: ESCRT-0, -I, -II, and -III, and VPS4-Vta1 [20]. ESCRT-III, the final complex in the MVBs degradation pathway, provides the core machinery that mediates membrane deformation and fission events during MVB biogenesis [21]. ESCRT-I and -III components have been found to localize to the midbody, and ESCRT-III components have been proposed to mediate membrane fission at the end of cytokinesis [22,23,24,25]. The vacuolar sorting protein Snf7 is an essential cellular component of ESCRT–III complex in multiple organisms that is involved in the sorting of transmembrane proteins en-route to lysosomal degradation through the endosomal-autophagic pathway [26]. Previous studies have demonstrated *Snf7* is an effective RNAi target for the control of pests, such as *D. v. virgifera* LeConte [7,26,27] and the southern corn rootworm *D. undecimpunctata howardi* [28]. The first commercially available genetically modified maize variety, MON87411, which includes an RNAi trait targeting *D. v. virgifera Snf7* against *D. v. virgifera*, was deregulated in the US in 2017 [29], and although the function of the Snf7 protein is conserved, suppression of the *Snf7* gene in *D. v. virgifera* via dsSnf7 is sequence-specific [30].

In this study, we evaluated the potential of the target gene *HvSnf7* in the control of *H. vigintioctopunctata*. Specifically, we (1) investigated expression patterns of *HvSnf7* across developmental stages and among tissues; (2) tested effects of oral ingestion of synthesized dsHvSnf7 concentrations on in vitro neonate survival rates and *HvSnf* gene expression; (3) quantified effects on larval and adult survival rates of ingestion of bacterially expressed dsHvSnf7; and (4) explored effects on ultrastructural changes to the larval midgut of ingestion of dsHvSnf7.

## 2. Materials and Methods

### 2.1. Insects

*Henosepilachna vigintioctopunctata* was collected from *Solanum nigrum* (L.) in April 2018 at the South China Agricultural University campus, Guangzhou City [31] and reared and maintained on detached *Solanum melongena* leaves in petri-dishes in an incubator set at 25 ± 1 °C, 14 L: 10 D, and 80% RH, before and during experiments.

### 2.2. Sample Preparation for Analysis of Hvsnf7 Expression

Samples of *H. vigintioctopunctata* at the egg, instar (×4), pupal, and adult (female and male) life history stages were collected as described in our recent study [31]. Tissue, comprising cuticle, fat body, midgut, and malpighian tubule, was dissected from 4th instar larvae, as described previously [31]. Each experiment was replicated three times. All the samples were quickly frozen in 1.5 mL tubes by liquid nitrogen, and stored at −80 °C before the total RNA extraction.

### 2.3. RNA Extraction and cDNA Synthesis

Total RNAs were isolated using TRIzol reagent (Invitrogen, Carlsbad, CA, USA) as previously described in our recent study [32]. A NanoDrop One^C^ spectrophotometer (Thermo Fisher Scientific, Waltham, MA, USA) was used to ascertain RNA concentration. First-strand cDNA was synthesized from 1.0 μg of total RNA in 20 μL volume using the PrimeScript reverse transcriptase (RT) kit (containing gDNA Eraser, Perfect Real Time, TaKaRa, Dalian, China) according the manufacturer’s recommendations. The cDNA was diluted 10-fold for the subsequent general polymerase chain reaction (PCR), reverse transcriptase-quantitative polymerase chain reaction (RT-qPCR), and dsRNA synthesis experiments.

### 2.4. dsRNA Preparation

Specific primers containing a T7 promoter sequence were used to generate dsRNAs against *HvSnf7* and green fluorescent protein (*GFP*) (Table 1). PCR products, which were purified using a Universal DNA Purification Kit (TIANGEN, Beijing, China), were used as a template to generate dsRNA using a T7 MEGAscript kit (Thermo Fisher Scientific) following the manufacturer’s protocol. Synthesized dsRNAs were suspended in ddH_2_O, and size and integrity of the purified dsRNAs were confirmed by loading the suspension onto a 1.5% agarose/Tris-acetate-EDTA (TAE) gel stained with GoldView I. Concentrations of purified dsRNAs were measured using a NanoDrop One^C^ spectrophotometer (Thermo Fisher Scientific), and the purified dsRNAs were stored at −20 °C prior to use.

### 2.5. Effects of Oral Ingestion of dsRNAs on Neonate Development and HvSnf Gene Expression

To assess effects of dsRNAs on larvae development, 12-mm diameter leaf-discs of *S. melongena* were immersed in one of three concentrations of dsHvSnf7 (5, 10, and 50 ng/µL) or 50 ng/µL of dsGFP for 10 s and supplied to five replicate Petri dishes containing 10 neonate larvae for 2 d; then fresh, untreated leaves were provided ad libitum for an additional 8 d. Larval mortality and developmental stages were recorded daily

To test for effects on *HvSnf* gene expression, neonate larvae were treated with either 10 ng/µL of dsHvSnf7 or 10 ng/µL of dsGFP; three replicates of 10 individuals were collected from each treatment after 2 and 4 d, flash-frozen in liquid nitrogen, and stored at −80 °C in 1.5-mL centrifuge tubes prior to total RNA extraction for analysis of *HvSnf* gene expression.

#### HvSnf Gene Expression Analysis

We analyzed *HvSnf* gene expression using RT-qPCR, using a reaction mixture and program that have been described previously [32] and gene-specific primers listed in Table 1. The reference genes *RPS18* and *RPL13* were used as internal controls [31] and relative quantification of *HvSnf7* was performed using the 2^−ΔΔ*C*t^ method [33].

### 2.6. Effects of Bacterially Expressed dsHvSnf7 on H. vigintioctopunctata Mortality

#### 2.6.1. dsRNA Synthesis in Bacteria

Bacterially expressed dsHvSnf7 and dsGFP were synthesized as previously described [8] using gene-specific markers (Table 1). PCR products were reorganized into a linearized L4440 vector used the Trelief SoSoo Cloning kit (Ver.2, TSINGKE, Beijing, China), following the manufacturer’s recommendations. Then, the L4440 vector containing the PCR products was transformed into competent HT115 (DE3) cells, strain of RNase III deficient *Escherichia coli*, and individual colonies were inoculated and grown until cultures reached an OD600 value of 0.5–0.8, when they were induced to express dsRNA by the addition of IPTG to a final concentration of 1mM. The expressed dsRNA was centrifuged at 5000× *g* for 10 min, and the bacteria cells were resuspended in an equal original culture volume of ddH_2_O.

#### 2.6.2. Effects on *H. vigintioctopunctata* Mortality

Bacterially expressed dsRNAs were administered, via *S. melongena* leaves in Petri dishes, to five replicates of 10 individuals of 1st and 3rd instar larvae and adults that had been starved for 3 h. We immersed 12-mm diameter leaf-discs of *S. melongena* in bacteria-expressed dsRNA solution for 10 s; the discs were then place on filter paper and air dried at room temperature for 1 h. Two leaf-discs were fed to 1st instar larvae, while 3rd instar larvae and adults were fed 20 leaf-discs; leaf-discs treated with dsRNA were replenished daily for two consecutive days, and from the third day onwards, insects were supplied with equal amounts of untreated fresh leaves. Assays continued for 10, 12, and 14 d for the 1st and 3rd instar larvae, and adults, respectively.

In a separate experiment, we placed three replicates of 10× 1st instar larvae, 5× 3rd instar larvae, and 5× adults to leaves of *S. melongena* plants that had been sprayed with bacterially expressed dsRNA for 1 h; each leaf was covered with a mesh bag to prevent the escape of the insects and the plants were placed in a ventilated cage (60 × 40 × 80 cm) under field-realistic environmental conditions (21–32 °C). After 5 d, mortality was recorded and the consumed leaves were photographed.

### 2.7. Analysis of Larval Midgut Ultrastructure

We treated 2nd instar larvae with 10 ng/µL of in vitro synthesized dsRNAs or 10 ng/µL of dsGFP for two consecutive days. On the 5th day, 10 surviving individuals were collected from each treatment and dissected to obtain the midguts.

The midguts were cut into smaller pieces (0.2-mm long) and prepared using fixing, embedding, and slicing methods previously described [34]. Samples were observed using a Talos L120C transmission electron microscope (Thermo Fisher Scientific) and images were recorded using an AMT digital image capturing system at optimal magnification (range of ×1600 to 5300).

### 2.8. Data Analysis

One-way analysis of variance (ANOVA) was used to compare *HvSnf7* expression levels across developmental stages and among tissue types, and to test for treatment effects on levels of *HvSnf7* gene expression at each time point and on larvae and adult mortality on the living plants. Means were compared using Tukey’s test at *p* < 0.05. Variation in target genes between the control and treatment was tested using ANOVA (Breslow pairwise comparison, *p <* 0.05). Survival curves, based on larval and adult mortality, were created using the Cox regression program. Proportional data were arcsine square root-transformed prior to analysis and data were analyzed using SPSS (v. 21.0; IBM Corp., Armonk, NY, USA).

## 3. Results

### 3.1. Temporal and Tissue Expression of HvSnf7

**Figure 1:**
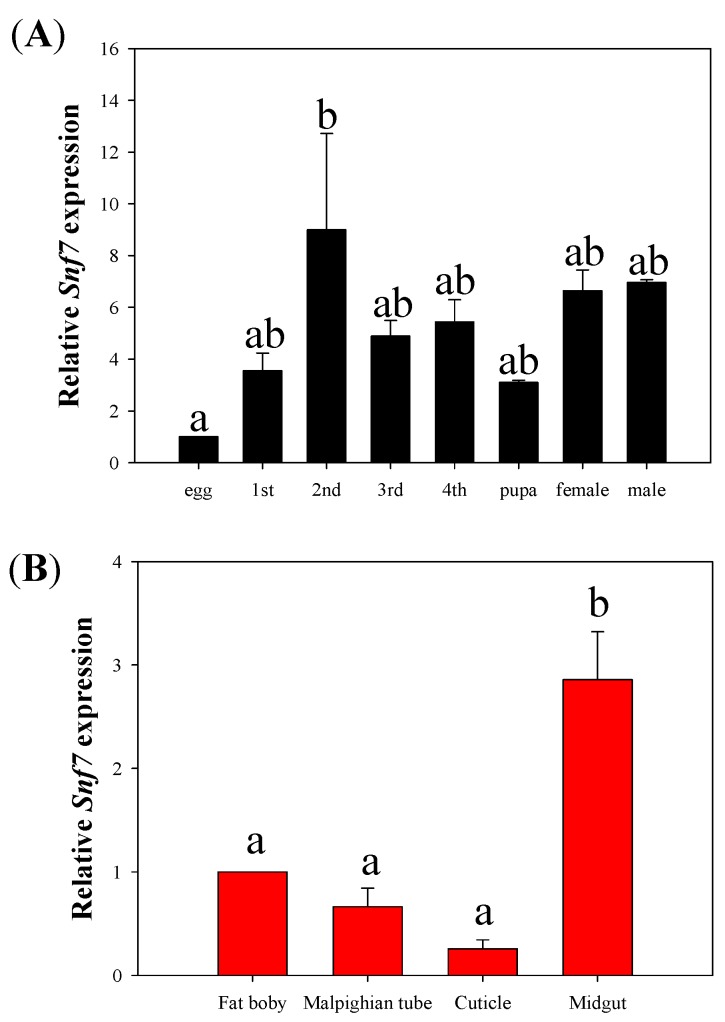
Expression patterns of *HvSnf7* across different developmental stages (**A**) and among tissues (**B**) in *H. vigintioctopunctata*. Data are means + starndard error (SE) and different letters indicate differences in gene expression at *p* < 0.05.

The results showed differences in *HvSnf7* expression across development stages (F_7,16_ = 3.155, *p* = 0.027) and among tissue types (F_3,8_ = 20.694, *p* < 0.0001), where lowest and greatest levels of expression were at the egg and 2nd instar stages, respectively; there were no differences in levels of gene expression among the other development stages (Figure 1A). Levels of expression of *HvSnf7* were greater in the midgut than in other tissues (Figure 1B).

### 3.2. Effects of Consumption of In Vitro Synthesized dsHvSnf7

**Figure 2:**
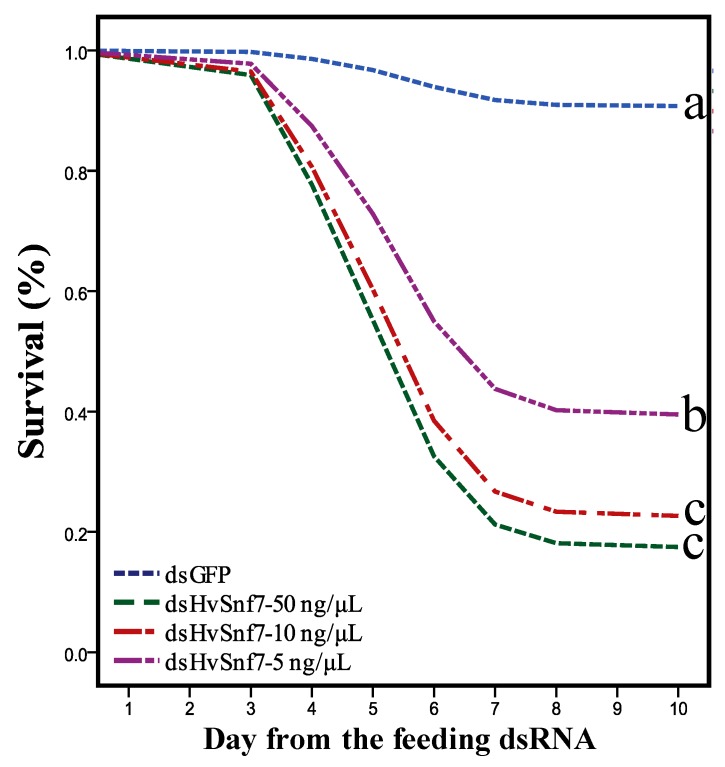
Impact of *HvSnf7* silencing using in vitro synthesized dsRNAs on *H. vigintioctopunctata* survival over 10 d. Different letters indicate treatment differences at *p* < 0.05.

Oral consumption of all concentrations of dsHvSnf7 led to greater overall levels of mortality after 10 d (χ^2^ = 84.235, *df* = 3, *p* < 0.0001), where there were 22.8, 16.8, and 10.0-fold increases in mortality following consumption of 50 ng/µL (*p* < 0.0001, Exp (B) = 22.767), 10 ng/µL (*p* < 0.0001, Exp (B) = 16.768), and 5 ng/µL (*p* < 0.0001, Exp (B) = 9.982) of dsHvSnf7 (Figure 2). Mortality was greater at 50 and 10 ng/µL of dsHvSnf7 than at 5 ng/µL (F_3,196_ = 67.621, *p* < 0.0001) (78, 70, and 54%, respectively) after 10 d.

**Figure 3:**
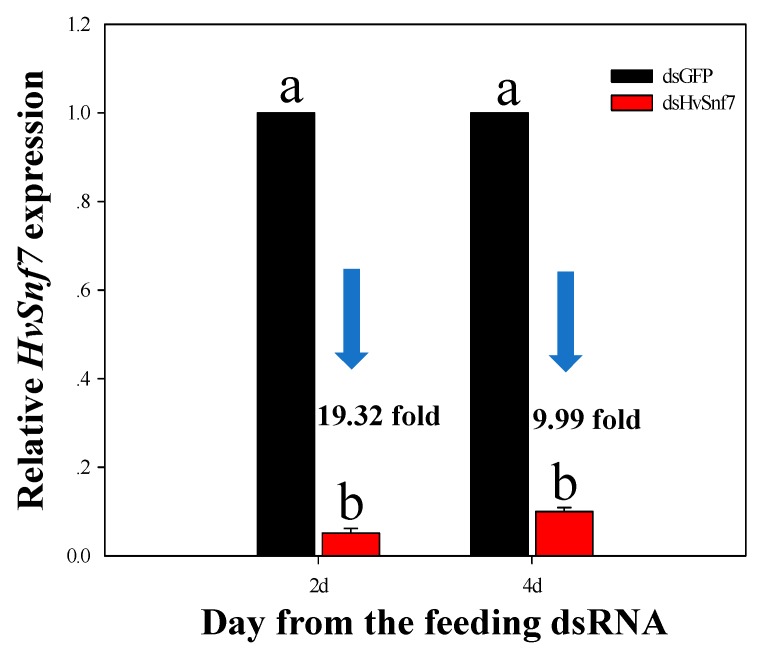
Expression of *HvSnf7* in *H. vigintioctopunctata* at 2 and 4 d after ingestion of in vitro synthesized dsHvSnf7 and dsGFP (10 ng/μL). Transcript levels of *HvSnf7* in dsGFP control larvae at each time point were set to 1. Data are means +SE and different letters indicate differences among treatments at *p* < 0.05.

Levels of *HvSnf7* expression were suppressed at days 2 (F_1,4_ = 8186.122, *p* < 0.0001) and 4 (F_1,4_ = 10947.665, *p* < 0.0001), where levels were suppressed 19.32 and 9.99-fold, respectively (Figure 3).

### 3.3. Impacts of Ingestion of Bacterially Expressed dsHvSnf7 on H. vigintioctopunctata Survival

**Figure 4:**
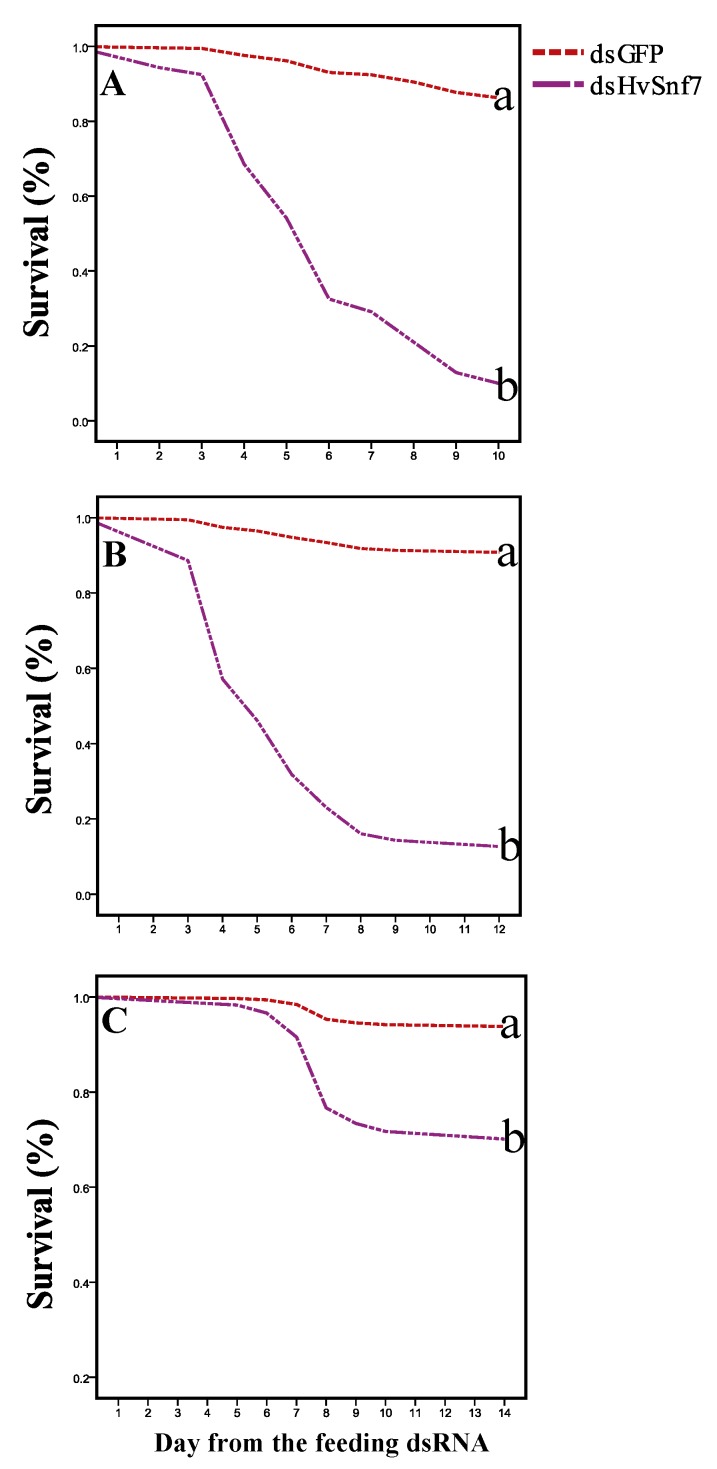
Impact of *HvSnf7* silencing on survival rates of 1st instar larvae (**A**), 3rd instar larvae (**B**), and adults (**C**) fed on detached leaves treated with bacterially expressed dsHvSnf7 at 10, 12, and 14 d, respectively. Different letters indicate treatment differences at *p* < 0.05.

We found that 1st and 3rd instar larvae, and adults began to die on the 2nd, 3rd, and 5th days after consumption of bacterially expressed dsHvSnf7 that caused 98% (*p* < 0.0001, Exp (B) = 21.452), 88% (*p* < 0.0001, Exp (B) = 15.624), and 60% (*p* = 0.007, Exp (B) = 5.537) mortality, respectively, by the end of the experiment (after 10, 12, and 14 d, respectively) (Figure 4). Compared with the control, ingestion of bacterially expressed dsHvSnf7 led to 21.452, 15.624, and 5.537-fold increases in mortality for 1st and 3rd instars, and adults, respectively.

**Figure 5:**
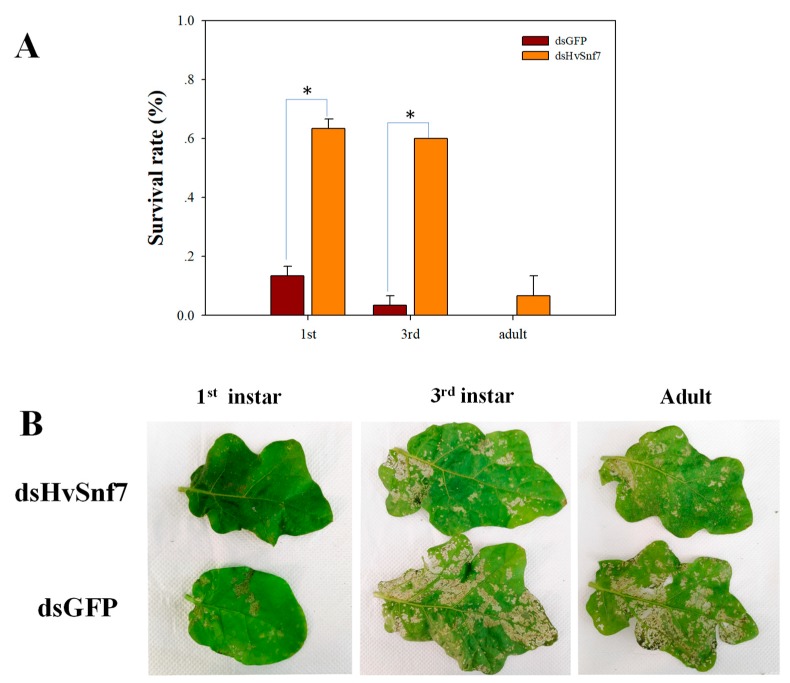
Impact of *HvSnf7* silencing on survival rates of larvae and adults fed on living plants treated with bacterially expressed dsHvSnf7 (**A**) and associated damage caused to leaves (**B**). * Treatment differences at *p* < 0.05.

We also found mortality effects for 1st (77%; F_1,4_ = 180.500, *p* < 0.0001) and 3rd (53%; F_1,4_ = 45.000, *p* = 0.003) instars feeding on *S. melongena* plants treated with bacterially expressed dsHvSnf7 after 5 d; however, there were no effects of mortality on adults (13%; F_1,4_ = 4.000, *p* = 0.116) (Figure 5A). Plants treated with dsHvSnf7 resulted in lower levels of *H. vigintioctopunctata* leaf damage (Figure 5B).

### 3.4. HvSnf7 Silencing Effects on Larval Midgut Ultrastructure

**Figure 6:**
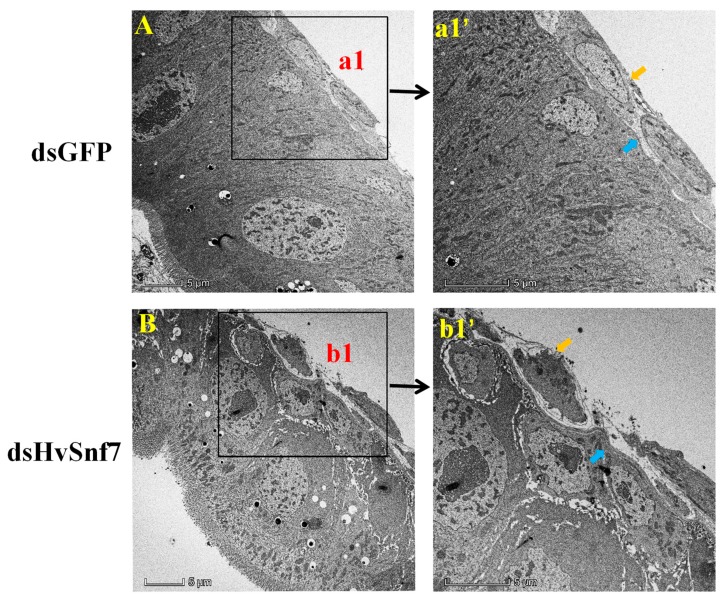
*HvSnf7* RNAi-mediated ultrastructural changes in larval midguts following ingestion of in vitro synthesized dsHvSnf7 and dsGFP (10 ng/μL). a1’ and b1’ are for the magnified view of a1 and b1, respectively. Blue indicates cell basement membrane; yellow indicates cell peritoneal membrane.

Electron microscopy of dsGFP-treated enterocytes revealed the presence of intact basement and peritoneal membranes; however, silencing of *HvSnf7* led to losses in integrity of enterocyte membranes. There was rupturing of the cell basement membrane, and boundaries between the cell internal chambers were damaged in dsHvSnf7-treated enterocytes. Ubiquitinated integral membrane proteins could not be sequestered by the fusion of MVB to autophagosomes and lysosomes; thus, membrane integrity was destroyed, and membrane receptor proteins and endocytosed materials, which were not subject to autophagic degradation, induced greater amounts of impurities in the cytoplasm (Figure 6).

## 4. Discussion

This study demonstrated the effects of RNAi on *H. vigintioctopunctata* larvae and adults exposed to dsRNA, by feeding in vitro synthesized and bacterially expressed dsHvSnf7 on detached leaves and living plants. Knockdown of *HvSnf7* caused high levels of mortality in *H. vigintioctopunctata*, indicating its potential use as an RNAi target gene for the control of this insect pest.

In insects, *Snf7* functions as a part of the ESCRT pathway that plays a crucial role in cellular housekeeping by internalization, transport, sorting, and lysosomal degradation of transmembrane proteins [20]. It has previously been shown that malfunctioning of these cellular processes in midgut and body fat tissues triggered by *Snf7* RNAi were the key drivers of mortality in *D. v. virgifera* [26], and the critical function of *Snf7* in insect midgut cells was confirmed by changes in *D. v. virgifera Snf7* larval enterocytes ultrastructure that underpin the conserved essential function of the ESCRT pathway in autophagy and membrane stability in other organisms [34]. Indeed, our results revealed that *Snf7* was highly expressed in the midgut of *H. vigintioctopunctata*, and electron microscopy of enterocytes revealed that dsHvSnf7 plays an important role in membrane stability. Specifically, cell homeostasis and integrity in dsGFP-treated enterocytes indicated that the autophagic degradation pathway continued to function properly; however, the structures of multi-lamellar bodies and macroautophagic complex in the midgut of dsHvSnf7-treated larvae were not detected, in contrast to a previous study [34]. The possible reasons for this difference include treatment duration, dsRNA dose, stage of insects, and study species.

Previous studies have shown large differences in RNAi efficiency among insect species [5,35,36]. RNAi-mediated silencing effects are efficient and systemic in coleopteran insects [7,8,15], and similarly, we found high levels of RNAi effects in *H. vigintioctopunctata,* even under the in vitro synthesized dsRNA concentration of 5 ng/µL. Therefore, there is great potential for the control of *H. vigintioctopunctata* using RNAi. We also found there was no difference in mortality effects of in vitro synthesized dsHvSnf7 concentrations of 50 and 10 ng/µL, indicating saturation of RNAi machinery was between 5 and 10 ng/µL.

This study provides evidence for the efficient induction of RNAi using bacteria to deliver dsRNA for the control of *H. vigintioctopunctata*, because bacterially produced dsRNAs inhibited growth and caused higher mortality in larvae and adults fed on detached leaves and living plants, resulting in improved plant protection. It is known that, prior to ingestion, dsRNA must be stable to generate an RNAi response in insects; however, factors, such as UV radiation and presence of microorganisms, degrade dsRNA in the environment, thus, it is possible that dsRNA modification or embedding by liposomes or nanoparticles may solve the problem of dsRNA degradation to improve RNAi efficiency [36]. Therefore, ensuring the stability of dsRNA in the environment should increase field control by dsHvSnf7 of larval and adult *H. vigintioctopunctata*.

One concern related to the use of dsRNA in pest management is the unintended effects on non-target organisms. One recent study evaluated the activity spectrum of dsSnf7 against *D. v. virgifera* for 14 insect species belonging to 10 families from four orders, and found it was narrow, with species taxonomically related to the target organism being relatively more susceptible [30]. Before dsHvSnf7 is used for field control of *H. vigintioctopunctata,* closely related non-target species that feed on Solanaceae plants and are exposed to insecticidal dsRNA should be assessed for potential risks of *H. vigintioctopunctata*-active dsHvSnf7 [37].

## 5. Conclusions

In summary, we identified and characterized an *HvSnf7* gene in *H. vigintioctopunctata* and showed its silencing using RNAi caused high levels of mortality and inhibited growth. Our findings confirmed our hypothesis that *HvSnf7* may be a potential target gene for RNAi-based control of the pest *H. vigintioctopunctata*.

## Figures and Tables

**Table 1:** Primers used for this study.

Gene	Primer Sequences (5′–3′)
HvSnf7-RT-qPCR-F	CAGAGAGGAACACTAGAGGAA
HvSnf7-RT-qPCR-R	GGTCAACGTTCATGTGTTTATG
dsHvSnf7-F	TAATACGACTCACTATAGGGACCCTTACAACCCTTGAATTAC
dsHvSnf7-R	TAATACGACTCACTATAGGGCTTCATCATCTTCCACTGCTT
dsGFP-F	TAATACGACTCACTATAGGGCTTGAAGTTGACCTTGATGCC
dsGFP-R	TAATACGACTCACTATAGGGTGGTCCCAATTCTCGTGGAAC
L4440-HvSnf7-F	ATCATCGATGAATTCACCCTTACAACCCTTGAATTAC
L4440-HvSnf7-R	TTCCTGCAGCCCGGGCTTCATCATCTTCCACTGCTT
L4440-GFP-F	ATCATCGATGAATTCCTTGAAGTTGACCTTGATGCC
L4440-GFP-R	TTCCTGCAGCCCGGGTGGTCCCAATTCTCGTGGAAC
